# Isolation, Characterization, and Optimization of Antimicrobial Proteinaceous Compounds Produced by *Bacillus safensis* Isolated From Rhizosphere Soil Samples in Bangladesh

**DOI:** 10.1155/ijm/3189187

**Published:** 2026-07-01

**Authors:** Fatema Tuz Jubyda, Mohammed Ayaz, Shyama Prosad Moulick, Firoz Ahmed, Md. Anowar Khasru Parvez

**Affiliations:** ^1^ Department of Microbiology, Jahangirnagar University, Savar, Dhaka, Bangladesh, juniv.edu; ^2^ BCSIR Dhaka Laboratories, Bangladesh Council of Scientific and Industrial Research (BCSIR), Dhaka, Bangladesh, bcsir.gov.bd; ^3^ BCSIR Rajshahi Laboratories, Bangladesh Council of Scientific and Industrial Research (BCSIR), Rajshahi, Bangladesh, bcsir.gov.bd

**Keywords:** antimicrobial proteinaceous compound (APC), *Bacillus safensis*, soil bacteria

## Abstract

*Bacillus* species are widely distributed in nature and are recognized for their capacity to synthesize a wide range of antimicrobial compounds effective against multidrug‐resistant bacterial and fungal pathogens, highlighting their potential as alternatives to conventional antibiotics. This study focused on the isolation of *Bacillus* spp. from rhizosphere soil capable of producing antimicrobial compounds effective against both gram‐positive and gram‐negative bacteria, and the optimization of different parameters to enhance their production. Of rhizosphere‐soil samples collected from eight different sites in Dhaka city, 96 bacterial isolates were selected to evaluate their antimicrobial activity. Twenty‐seven isolates were preliminarily identified as *Bacillus* spp. based on their morphological characteristics and biochemical reactions. Five isolates were found to produce inhibitory compounds having antimicrobial properties. The highest antimicrobial compound production was observed at pH 7 and 30°C, rather than at alkaline or acidic pH (*p* value = 0.04, < 0.05). Sequence analysis of the 16S rRNA gene of three significant isolates producing antimicrobial compounds revealed that isolates S02b and S03b had 100% sequence similarity to *Bacillus safensis*, and S04b had 100% similarity with *Bacillus pseudomycoides*. Whole‐genome sequencing revealed that isolate S02b harbors biosynthetic gene clusters (BGCs) similar to *schizokinen* (60%), *fengycin* (53%), *bacilysin* (85%), *bacillibactin* (80%), and *lichenysin* (92%), indicating specialization in peptide‐based secondary metabolite production. It also identified one RiPP and three putative bacteriocin clusters, with area of interest (AOI) 1 matching sactipeptide synthetase (BmbF), amylocyclicin, and UviB, and AOI 2 matching UviB. Thin layer chromatography (*R*_*f*_ values: 0.45–0.52), the absorbance peak in UV spectra (210–220 nm), and protease sensitivity confirmed the proteinaceous nature of the extracted antimicrobial compounds. The antimicrobial proteinaceous compound (APC) from isolate S02b was nonhemolytic and stable at heat, pH, surfactant, food‐grade metal salt solvent, and organic acid. The scanning electron microscopy image revealed that APCs exhibited significant antimicrobial activity against *Staphylococcus aureus*, suggesting that APCs could be a promising candidate for new antibiotics.

## 1. Introduction

Infectious diseases are responsible for one of the highest global burdens of premature death [1], and finding novel medications to fight microbial pathogens is crucial [2]. In 2019 alone, 4.95 million deaths were attributed to bacterial antimicrobial resistance (AMR) worldwide [3]. Overuse of traditional antibiotics in agriculture and animal husbandry, particularly in developing nations, raises concerns about the promotion of AMR in the environment [4, 5]. Alternatives are urgently needed, and antimicrobial proteinaceous compounds (APCs), such as antimicrobial peptides (AMPs), with broad‐spectrum activity offer a possible solution to conventional strategies [6].

AMPs are commonly observed in nature, and over 3000 molecules have been reported [7]. They can affect the innate systems of not only animals and plants, but also viruses [8]. AMPs′ potential has been expanded to treating HIV infection, as well as cancer and tumors [9–11]. Since AMPs primarily kill microbes by targeting their membranes through pore‐forming mechanisms, they can be an effective strategy to combat the drug‐resistant pathogens causing infectious diseases [6].

Members of the genus *Bacillus* are capable of producing a large number of APCs, synthesized nonribosomally and ribosomally [12, 13]. Nonribosomally synthesized *Bacillus*‐derived peptide antibiotics include bacitracin, gramicidin, surfactin, tyrocidine, fengycins, and iturins [14]. Ribosomally synthesized APCs are lipopeptides, bacteriocins, glycopeptides, and cyclic peptides. These bacteriocins are secreted into the extracellular matrix during their metabolism and can inhibit the growth of closely related species or species from other phyla or domains. *Bacillus* strain was confirmed to be an efficient producer of peptide antibiotics, demonstrating strong potential for application as a biocontrol agent [15]. *Bacillus safensis* APC 4099 exhibited broad‐spectrum antimicrobial activity against gram‐positive foodborne pathogens and food‐spoilage fungi, which is attributed to the production of AMPs and secondary metabolites [16]. The strain was found to produce bacteriocins, including pumilarin and plantazolicin, as well as secondary metabolites such as pumilacidin A, bacilysin, and bacillibactin [16].

The food industry takes advantage of the antimicrobial activity of APCs, such as AMPs, as a preservative because these peptides can be enzymatically degraded and do not affect the human body [17]. Many *Bacillus* spp. are known to produce at least one APC as part of their defense mechanism, and their exploration in soil microbiomes is a promising arena for isolating APCs [18].

The aim of this study was to identify *Bacillus* spp. that produce APCs and optimize their growth conditions, including temperature, pH, and incubation period, for enhanced APC production.

## 2. Materials and Methods

The present study was designed to identify and characterize antimicrobial compound‐producing *Bacillus* spp. from soil samples collected in Savar, Dhaka City, with the optimization of different parameters (temperature, pH, and incubation period) for enhanced antimicrobial compound production.

### 2.1. Collection of Soil Samples

Rhizosphere soil samples were collected from eight locations across different parts of the Savar area based on the region′s plantation coverage and vegetation diversity. Geographic locations of soil sampling sites and their associated soil textural classifications are provided in Table S1. Rhizosphere soil was collected from a depth of 5–10 cm to capture soil under active rhizosphere influence with a relatively stable microbial community, while minimizing surface contamination and environmental fluctuations. Samples were aseptically collected using a sterile soil probe [19], stored in ziplock bags, and transported at 4°C to the Novel Exploration of Therapeutics (NEXT) Biosciences Laboratory, Department of Microbiology, Jahangirnagar University.

### 2.2. Sample Preparation

After sample collection, the serial dilution method was followed. About 1 g of soil sample was diluted into 9 mL of sterilized distilled water and subsequently diluted up to 10^5^ times. Each diluted sample was spread on a nutrient agar plate containing cycloheximide 0.3 g/20 mL to prevent fungal contamination, and the plate was incubated at 37°C for 24 h [20].

### 2.3. Identification of *Bacillus* spp.

Isolates were identified based on their morphological and biochemical characteristics according to Bergey′s Manual of Determinative Bacteriology [20]. Gram staining was performed, and the results were observed under a light microscope. Isolates were subjected to IMViC tests (indole production, methyl red [MR], Voges–Proskauer [VP], and citrate utilization), catalase tests, and carbohydrate fermentation tests. Confirmation was done by using the VITEK 2 system (bioMérieux, Inc., Hazelwood, Missouri, United States). The isolates were stored at −20°C for subsequent analysis.

### 2.4. Test Host Strains for the Study

ATCC strains of gram‐positive and gram‐negative bacteria were used as hosts to assess the antimicrobial compound production of *Bacillus*. Gram‐negative hosts included *Pseudomonas aeruginosa* ATCC 27853, *Escherichia coli* ATCC 25922, MDR clinical isolate of *E. coli* JU‐EC1, *Shigella dysenteriae* JU‐SD1, and *Salmonella enteritidis* ATCC 13076. Gram‐positive hosts included *Listeria monocytogenes* ATCC 13932, *Staphylococcus aureus* ATCC 25923, and *Bacillus cereus* ATCC 11778. ATCC reference strains were supplied by the NEXT Biosciences Laboratory, Department of Microbiology, Jahangirnagar University, whereas the MDR clinical isolate *E. coli* JU‐EC1 and *S*. *dysenteriae* JU‐SD1 were obtained from the Environmental Health and Synthetic Biology Laboratory, Department of Microbiology, Jahangirnagar University.

### 2.5. Isolation of *Bacillus* spp. Having Antimicrobial Properties

Isolated colonies were inoculated individually in Luria–Bertani (LB) broth and incubated at 37°C for 72 h [20]. Samples were collected and centrifuged at 12,000 rpm for 10 min at each 24‐h interval to obtain cell‐free supernatant (CFS). The CFS was stored at 4°C and subsequently subjected to the agar well diffusion method to evaluate the antimicrobial properties of CFS extracted from study isolates. Twenty‐four‐hour fresh cultures of test host strains were inoculated into LB broth, where the turbidity of the cultures was adjusted to 0.5 McFarland, corresponding to approximately 1.5 × 10^8^ CFU/mL. The lawn of test strains was prepared, and wells (6 mm in diameter) were created on the nutrient agar plates using a sterile cork borer. Sixty microliters of CFS extracted from each isolate was poured into each well, and the plates were incubated at 37°C for 24 h. After 24 h, the zones of inhibition (ZOIs) were observed and measured using a transparent metric ruler and recorded in millimeters [21]. The results were compared with those obtained using ciprofloxacin (5 *μ*g) as positive control. Isolates that exhibited ZOI against test strains underwent subsequent steps to extract antimicrobial compounds.

### 2.6. Antimicrobial Compound Production by *Bacillus* spp.

#### 2.6.1. Preparation of Bacterial Inoculum

Inoculum was prepared in LB broth by inoculating isolated *Bacillus* spp. having antimicrobial activity separately and incubated at 30°C for 72 h.

### 2.7. Media Optimization

Four synthetic media (a–d) were used for better production of antimicrobial compound with slight modification, (a) basal medium containing (g/L): Na_2_HPO_4_.2H_2_O 7.9, KH_2_PO_4_ 3.0, NaCl 0.5, NH_4_Cl 1.0 (pH 7.2); with glucose, lactose, peptone, and yeast extract, 0.5% each of them added to the medium to know the effect of carbon and nitrogen sources [12]; (b) yeast‐rich medium containing (g/L): peptone 25, sucrose 20, yeast extract 8.0, KH_2_PO_4_ 2.0, MgSO_4_.7H_2_O 0.045, and trace elements (MnSO_4_.H_2_O 0.004, CuSO_4_.7H_2_O 0.005, and ZnSO_4_ 0.014) [22]; (c) synthetic medium containing (g/L): L‐glutamic acid 5.0, KH_2_PO_4_ 0.5, K_2_HPO_4_ 0.5, MgSO_4_.7H_2_O 0.2, MnSO_4_.H_2_O 0.01, NaCl 0.01, FeSO_4_.7H2O 0.01, CuSO_4_.7H_2_O 0.01, CaCl_2._H2O 0.015, glucose 10, and pH 7 [23]; and (d) minimal medium containing (g/L): MnSO_4_.H_2_O 0.0017, ZnSO_4_ 0.0016, FeSO_4_ 0.02, NaCl 0.01, MgSO_4_ 0.6, KH_2_PO_4_ 0.14, K_2_HPO_4_ 2.2, CaCO_3_ 1.0, KCl 4.0, glucose 20, and pH 7.2 [24]. About 10% inoculum of each desired isolate was added to the flask containing 200 mL of synthetic media for antimicrobial compound production. Flasks were incubated at 30°C. Samples were taken up to 240 h at intervals of every 24 h and subsequently centrifuged to get CFS at 12,000 rpm for 10 min, which were sterilized through 0.2‐*μ*m filter paper. The production of the antimicrobial compound was evaluated using an agar well diffusion assay.

### 2.8. Optimization of Antimicrobial Compound Production

Temperature and pH are essential parameters for the successful production of antimicrobial compounds. The effect of the incubation period at different pH and temperatures on the maximum production of antimicrobial compounds was evaluated by adjusting these factors to optimize the production of the inhibitory substance. All assays were performed in triplicate, and the average values are presented.

#### 2.8.1. Optimization of Temperature

Different temperatures, including 30°C, 37°C, and 42°C, were used to produce better antimicrobial compounds. Isolates were incubated constantly for up to 240 h. Cultures were taken every 24 h and centrifuged to extract antimicrobial compounds. The antimicrobial activity of the inhibitory substance was evaluated by the agar well diffusion method against test host strains [25].

#### 2.8.2. Optimization of pH

Different strains of *Bacillus* spp. exhibit varying optimum pHs for the production of antimicrobial compounds. We selected three pH levels (5.5, 7, and 8) and three temperatures (30°C, 37°C, and 42°C), with a constant incubation period of up to 240 h. After every 24 h, cultures were collected and centrifuged at 12000 rpm for 10 min to extract antimicrobial compounds to optimize the production of these inhibitory compounds. The extracted compound was subjected to antimicrobial susceptibility testing using the agar well diffusion method to evaluate its antimicrobial activities [25]. The results were compared with those obtained using ciprofloxacin (5 *μ*g) as positive control.

### 2.9. Molecular Identification of Selected Study Isolates

Molecular‐level identification of selected study isolates was performed by amplifying the 16S rRNA gene by PCR using universal primers, and the amplified PCR products were sequenced. The following primers were used in this study:

Forward primer 27F: 5 ^′^‐AGAGTTGATCCTGGCTAAG‐3 ^′^

Reverse primer 1492R: 5 ^′^‐GGTTACCTTGTTACGACTT‐3 ^′^

PCR amplification was performed using a Biometra PCR Thermal Cycler (Analytik Jena, Jena, Germany). Thermal cycling conditions were as follows: initial denaturation at 95°C for 5 min, followed by 35 cycles of denaturation at 94°C for 30 s, annealing at 50°C for 40 s, and extension at 72°C for 1 min. The final extension was for 5 min at 72°C [26].

### 2.10. Sequencing of 16S rRNA Gene

The amplified PCR product was purified and sequenced. The sequences were analyzed and compared with reference sequences available in the GenBank database using the Basic Local Alignment Search Tool (BLAST).

### 2.11. Analysis of Sequence Data and Construction of Neighbor‐Joining Tree to Evaluate Their Evolutionary Relationship

The DNA sequences obtained from the NCBI nucleotide database were aligned using the MUSCLE program in MEGA 11. The neighbor‐joining tree was constructed using the Kimura two‐parameter model with 1000 bootstrap replicates [27].

### 2.12. Whole‐Genome Sequencing (WGS), Genome Assembly, Classification, and Annotation Analysis

Genomic DNA was extracted from the S02b using the Qiagen DNeasy Blood & Tissue Kit (Qiagen, #69504). The bacterial pellet was centrifuged at 14,000 rpm for 5 min to facilitate DNA purification. The quality of the extracted DNA underwent rigorous quality control assessment to ensure its suitability for WGS. DNA purity was verified using a Nanodrop (Thermo Fisher Scientific, ND‐1000) spectrophotometer, with a 260/280 ratio of 1.8 indicating high purity and minimal contamination, and a 260/230 ratio of 2.0, confirming negligible interference from other substances, such as carbohydrates or organic compounds. DNA concentration was quantified using a Qubit 4.0 Fluorometer, which indicated a satisfactory concentration. The Qubit working solution was prepared by combining Qubit Reagent with Qubit Buffer in a 1:200 ratio. Measurements were then conducted on both samples and standards in the appropriate assay tubes. For library preparation, the Illumina DNA Prep Reagent Kit (20060059) was used alongside an automated liquid handler (epMotion 5075). Postpooling, the library concentration was quantified using a Qubit 4.0 fluorometer, ensuring it was adequate for sequencing. Following the NextSeq System Denature and Dilute Libraries Guide (15048776), the pooled library was diluted to 4 nM. For denaturation, 5 *μ*L of this dilution was mixed with 5 *μ*L of 0.2 N NaOH and 5 *μ*L of 200 mM Tris‐HCl, pH 7.0. An addition of 990 *μ*L of HT1 buffer converted the mixture into a 20‐pM library solution. Subsequently, a 1.5‐pM library solution was loaded onto the NextSeq 2000. The sequencing process used fluorescently labeled nucleotides to decode the DNA sequence from the prepared libraries, using paired‐end 2 × 150 bp reads to capture detailed genetic data.

The raw sequencing data generated in this study were deposited in the NCBI Sequence Read Archive (SRA) under the BioProject accession PRJNA1337674. Raw sequencing data were evaluated using FastQC (v0.11) [28] and filtered to remove contaminants, adapters, and low‐quality sequences using Trimmomatic (v0.39) [29]. After trimming, the high‐quality reads were de novo assembled using SPAdes v3.15.4 [30]. Reads for S02b were submitted to PATRIC (https://www.patricbrc.org) for comprehensive genome analysis, which assembled the raw sequence reads and annotated the contigs using RASTtk [31]. Preassembled contigs were submitted to Bakta (https://bakta.computational.bio/) [32] for annotation and visualization, featuring interactive circular genome maps. Phylogenetic analysis was performed using the Type (Strain) Genome Server (TYGS) with the maximum‐likelihood method [33], and the resulting phylogenetic tree was visualized using iTOL (https://itol.embl.de/) [34]. For further confirmation of the isolate′s similarity, the average nucleotide identity (ANI) was calculated using FastANI [35] and compared with the genome of *Bacillus* retrieved from the NCBI database (https://www.ncbi.nlm.nih.gov/). Biosynthetic gene clusters (BGCs) responsible for the secondary metabolite production were identified using antiSMASH (https://antismash.secondarymetabolites.org/) [36] and BAGEL4 (http://bagel4.molgenrug.nl/) [37].

### 2.13. Hemolytic Activity

The hemolytic activity of the antimicrobial compound was examined on blood agar (5%–10% sheep blood) by following the agar well diffusion technique. Wells were made in the blood agar plates and incubated with 50 *μ*L of the extracted inhibitory compound in the well at 37°C for 24 h [38].

### 2.14. Identification of Antimicrobial Compounds by Thin Layer Chromatography (TLC)

TLC is used as a preliminary screening tool for the qualitative identification and confirmation of protein‐associated components prior to definitive analysis by advanced analytical techniques. It was conducted on a thin layer of aluminum sheet coated with absorbent material called TLC plate. Silica gel was used as a stationary phase. To apply sample spots, thin marks were made at the bottom of the plate using a pencil. The filtrate CFS containing antimicrobial compounds was spotted on the marked spots on the plate. The mobile phase (n‐butanol‐methanol‐water 3:1:1 *v*/*v*) was poured into the TLC chamber and to maintain equal humidity, moistened filter paper was placed in the mobile phase. The plate in the TLC chamber was placed, and it was closed with a lid. It was kept in such a way that the sample faced the mobile phase. The plate was immersed for the development of the spot. The sample spots were kept well above the level of the mobile phase. Once the spots were developed, the plate was taken out and dried. The TLC plate was sprayed with water and ninhydrin (0.2%) to determine the lipophilicity of the substance and the presence of amino acids in the sample [39].

### 2.15. Characterization of Antimicrobial Compound

To determine whether the antimicrobial activity of the inhibitory substance was due to a proteinaceous compound or the production of organic acids, the nature of the antimicrobial agent was assessed by measuring the pH and treating the sample with the enzyme, trypsin (1 mg/mL). The activity of the treated antimicrobial compound with trypsin was tested (1:1 *v*/*v*) against the test host strains, followed by measuring the ZOI [40].

### 2.16. Spectroscopic Characterization of Antimicrobial Compound

UV spectroscopy is used for preliminary peptide identification by detecting characteristic absorbance of peptide bonds (190–230 nm) and aromatic residues (260–280 nm), providing an initial assessment of the presence of peptide prior to detailed analysis [41]. The ultraviolet absorbance spectrum was recorded using a spectrophotometer (UV1800, Shimadzu, Japan) on a scan range of 200–400 nm.

### 2.17. Ammonium Sulfate Precipitation and Desalting of APCs

The APCs were precipitated from the CFS using ammonium sulfate 80% saturation (approximately 3.9–4.0 M at 0°C–4°C) [42]. Ammonium sulfate was slowly added to the CFS with continuous stirring for an additional 1–2 h at 4°C to achieve maximum APCs precipitation. The precipitates were then subsequently collected by centrifugation at 10,000 × g for 20 min at 4°C. The resulting pellets were resuspended in phosphate buffer saline and dialyzed against the same buffer at 4°C using dialysis column (Cobetter Briscale centrifugal filters) to remove residual ammonium sulfate.

### 2.18. Stability of the Antimicrobial Activity of APCs

The stability of the antimicrobial activity of the untreated APCs extracted from isolate S02b was evaluated against *S. aureus* under various conditions, including temperature, pH, surfactants, organic solvents, and salts.

#### 2.18.1. Effect of Temperature

Extracted APCs were incubated at different temperatures for 15 min: 50°C, 60°C, 70°C, 80°C, 90°C, and 100°C, and their stability was assessed under these different conditions [43].

#### 2.18.2. Effect of pH

The effect of pH on the antimicrobial activity of APC extracted from isolate S02b was examined at different pH levels using 0.1‐M buffers: citrate buffer at pH 3.0 and 5.0, phosphate buffer at pH 6.5, and Tris buffer at pH 8.0 and 10. After incubation with APC (1:1 *v*/*v*) at 37°C for 2 h, antimicrobial activity was checked against *S. aureus* [44].

#### 2.18.3. Effect of Surfactants

The extracted APC was incubated with 1.0% (*v*/*v*) of sodium dodecyl sulfate (SDS), Tween 20, Tween 80, and Triton X‐100 for 5 h at 37°C, and the antimicrobial activity was subsequently assessed [44].

#### 2.18.4. Effect of Organic Solvents, Compounds, and Food‐Grade Metal Salts

The APC was treated with different organic solvents and compounds, including ethyl alcohol, methanol, chloroform, isopropanol, phenol, DMSO, EDTA, n‐hexane, and dimethyl sulfonate, at a final concentration of 50% (*v*/*v*) and incubated at 30°C for 1 h before assessing antimicrobial activity. To evaluate the effect of food‐grade metal salts on APC activity, the compound was incubated with CuSO_4_, MgSO_4_, MnCl_2_, AgNO_3_, ZnSO_4_, FeSO_4_, and CaCl_2_ at a final concentration of 1.0 mg/mL for 1 h at 37°C, and activity was measured against *S. aureus*. Untreated APC and the metal salts at their respective concentrations were used as controls [26].

#### 2.18.5. Effect of Organic Acids

Various organic acids (tartaric acid, lactic acid, and citric acid) were used to treat the APC at a final concentration of 10 mg/mL, and the samples were incubated at 37°C for 5 h before antimicrobial activity was assessed.

### 2.19. Scanning Electron Microscopy (SEM) Study to Evaluate the Effect of APC Against the Test Host Strain *S. aureus*

SEM was used to examine changes or any rupture in the cell morphology of the test host strain. The preferred test strain, *S. aureus*, was grown overnight in LB media and treated with APC (*v*/*v*), and the untreated bacterial culture was considered as a control. The bacterial cells from both treated and untreated cultures were recovered by centrifugation at 6000 rpm, and the cells were washed twice with potassium phosphate buffer (50 mM, pH 7.0). The pellets were washed twice with potassium phosphate buffer and left overnight at 4°C. Then the pellets were washed twice with phosphate buffer and dehydrated by ethanol series (*v*/*v*) ranging from 30%, 40%, 50%, 60%, 70%, 80%, 90%, and 100% (*v*/*v*) and dried to critical point, coated with gold, and inspected with an S‐200C SEM. Results were compared with the standard pathogenic test host culture as a control.

### 2.20. Statistical Analysis

Data were compiled, tabulated, and analyzed under the research objectives. All statistical analyses were performed using IBM SPSS Statistics Version 25. Descriptive statistics were reported as frequency along with percentage. Pearson′s chi‐square test was used to test the association between categorical data. A two‐tailed *p* value < 0.05 was considered statistically significant.

## 3. Results

### 3.1. Morphological and Biochemical Identification of Study Isolates

**Figure 1:**
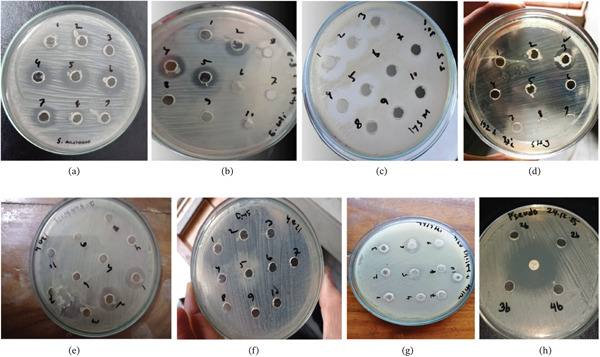
Antibacterial activity of extracted compounds against (a) *S. aureus*, (b) MDR *E. coli*, (c) *L. monocytogenes*, (d) *E. coli*, (e) *B. cereus*, (f) *S. dysenteriae*, (g) *P. aeruginosa*, (h) *P. aeruginosa* (with control ciprofloxacin, 5 *μ*g) after optimization of temperature and pH. The numbering denotes the ZOI of (a–g) antimicrobial compounds collected at 24‐h intervals: 1 corresponds to 24 h, 2 to 48 h, and subsequent numbers indicate successive time points.

**Figure 2:**
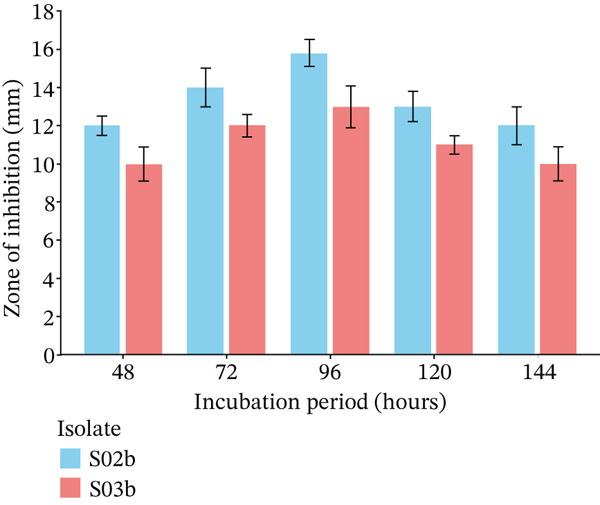
Antimicrobial activity of the antimicrobial compound produced from isolates S02b and S03b against the test strain MDR *E. coli.* The production of antimicrobial compounds from isolates S02b and S03b showed the highest ZOI against MDR *E. coli* at 30°C after 96 h of incubation; however, the production gradually decreased over time. The experiment was performed in triplicate, and error bars represent the mean ± standard deviation of the three independent replicates.

**Figure 3:**
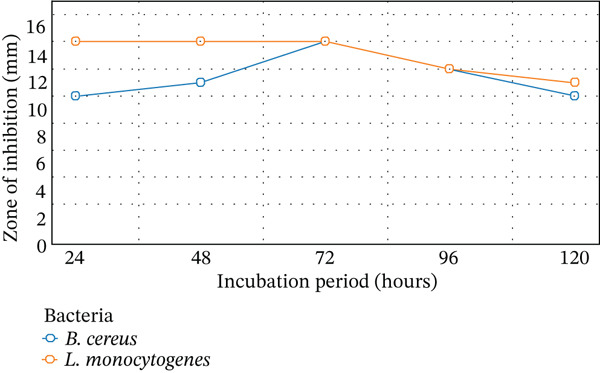
Antibacterial activity of extracted antimicrobial compound from isolate S02b at pH 5.5, 30°C. This isolate exhibited increased antimicrobial activity with extended incubation periods ranging from 24 to 72 h, followed by a decline in activity beyond 72 h against *L. monocytogenes*. Furthermore, the strain demonstrated a significant level of inhibitory compound production up to 216 h of incubation; however, production declined with further incubation against *B. cereus.*

Soil samples were collected from eight distinct rhizosphere locations in Savar, Bangladesh, and 27 *Bacillus* isolates were isolated based on their colony and morphological characteristics, biochemical tests, and the VITEK 2 isolation kit. Colonies presumptively identified as *Bacillus* spp. were large, whitish, and convex to irregular, with a dry appearance. Gram staining confirmed that 28% of the isolates (27/96) were gram‐positive rods. All presumptive isolates (27/27, 100%) were indole‐negative, catalase‐positive, and capable of starch hydrolysis. Most isolates were MR positive (24/27, 88%), whereas all were VP‐negative. Citrate utilization was observed in 15% of the isolates (4/27) (Figures S1, S2, and Table S2). In the agar well diffusion method, CFS of five isolates (*n* = 5/27, 18%) showed significant antimicrobial activity against study host bacteria, producing ZOIs greater than 9 mm (ZOI < 9 mm was considered nonsignificant) (Table 1 and Figure 1). Ciprofloxacin (5 *μ*g), used as the positive control, produced a ZOI of around 22 mm. Isolates S02b and S03b exhibited antimicrobial activity against three gram‐positive strains (*L. monocytogenes* ATCC 13932, *S. aureus* ATCC 25923, and *B. cereus* ATCC 11778) and three gram‐negative strains (*P. aeruginosa* ATCC 27853, *E. coli* ATCC 25922, and *S. dysenteriae* JU‐SD1), including MDR clinical isolate *E. coli* JU‐EC1, except *S*. *enteritidis* ATCC 13076 (Table 1). The highest level of antimicrobial compound production was observed at 30°C and at a neutral pH (pH 7), regardless of the different incubation periods, and the best result was obtained with synthetic medium (c) (Figures 2 and 3).

**Table 1:** Antimicrobial activity of study isolates at 30°C against test host strains at neutral pH.

*Bacillus* isolate	*Z* *o* *n* *e* of *i* *n* *h* *i* *b* *i* *t* *i* *o* *n* (*Z* *O* *I*) (*m* *m*) ± *S* *D* for *h* *o* *s* *t* *b* *a* *c* *t* *e* *r* *i* *a*							
	*S. aureus*	*E. coli*	MDR *E. coli*	*B. cereus*	*S. dysenteriae*	*L. monocytogenes*	*P. aeruginosa*	*S. enteritidis*
S01b	12.0 ± 0.0	—	—	13.0 ± 1.4	13.0 ± 3.0	11.3 ± 1.6	—	—
S02b	20.7 ± 2.5	13.3 ± 1.5	15.8 ± 1.6	9.7 ± 0.6	17.3 ± 2.1	9.0 ± 0.6	9.9 ± 0.8	—
S03b	17.7 ± 2.5	11.7 ± 1.5	13.2 ± 1.1	9.5 ± 0.7	12.0 ± 1.0	9.0 ± 1.0	9.2 ± 0.3	—
S04b	13.0 ± 1.0	19.3 ± 2.1	—	10.0 ± 0.0	—	11.3 ± 0.8	9.6 ± 0.4	—
S06b	10.0 ± 0.0	—	—	11.0 ± 1.0	—	—	—	—

We investigated the ZOI of isolates for different incubation periods. The production of antimicrobial compounds from isolates S02b and S03b showed the highest antimicrobial activity against MDR *E. coli* after 96 h of incubation at 30°C and neutral pH, with ZOI of 15.8 ± 1.6 mm and 13.2 ± 1.1 mm, respectively. However, the production declined progressively with an extended incubation period (Figure 2). Isolate S02b showed increasing production of antimicrobial compounds between 24 and 72 h of incubation at 30°C and pH 5.5, followed by a decline after 72 h against *L. monocytogenes*. Furthermore, the strain produced substantial levels of inhibitory compounds for up to 216 h of incubation; however, production decreased with longer incubation against *B. cereus* (Figure 3). The production of antimicrobial compounds was significantly higher at pH 7 compared with pH 5.5 and pH 8 (*p* = 0.04∗, < 0.05). Among significant isolates, only S02b exhibited ZOI at pH 5.5. At pH 8, both isolates S02b and S03b demonstrated reduced antimicrobial compound production, with ZOI values of less than 8 mm, regardless of the incubation periods.

The representative images showed ZOI against host bacteria in Figure 1. The numbering in Figure 1 represents the ZOI of antimicrobial compounds produced at 24‐h intervals: 1 corresponds to production of the compound after 24 h, 2 after 48 h, and subsequent numbers indicate production at successive time points.

### 3.2. Molecular Identification of Selected Study Isolates and Analysis of 16S rRNA Gene Sequencing

The full‐length 16S rRNA gene (1.5 kb) of the five *Bacillus* isolates with antimicrobial activity was amplified using universal primers. PCR amplicons of the 16S rRNA gene of three isolates (S02b, S03b, and S04b) were sequenced. BLAST homology search revealed that the isolates S02b and S03b had 100% nucleotide identity to that of *B*. *safensis*, particularly with *B*. *safensis* FO‐36b strain Y175 and *B*. *safensis* FO‐ZL8, respectively. The 16S rRNA gene sequence of strain S04b in this study showed 100% nucleotide identity to *Bacillus pseudomycoides* strain 1AL1.

### 3.3. Evolutionary Relationships of Taxa

**Figure 4:**
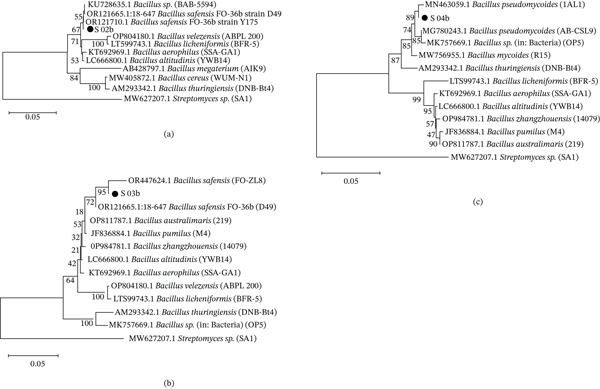
Phylogenetic relationship of soil isolate (a) S02b, (b) S03b, (c) S04b with other *Bacillus* spp., constructed using the Kimura two‐parameter model in MEGA11. Isolates S02b and S03b showed 100% nucleotide identity to that of *Bacillus safensis*, particularly with *B*. *safensis* FO‐36b strain Y175 and *B*. *safensis* FO‐ZL8, respectively. The sequence of the 16S rRNA gene of strain S04b in this study showed 100% nucleotide identity to *Bacillus pseudomycoides* strain 1AL1.

**Figure 5:**
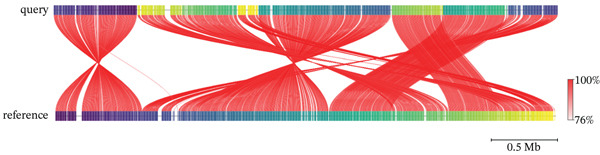
Genome sequencing data depicted the highest sequence similarity with *Bacillus safensis*, showing 96.09% nucleotide identity with the reference genome of *Bacillus safensis* (ASM824476v1). Each red line segment denotes a reciprocal mapping between the query and reference genome, indicating their evolutionary conserved regions.

The neighbor‐joining tree was constructed from 16S rRNA gene sequences of other soil *Bacillus* strains, confirming that isolate S02b belongs to the genus *Bacillus*. It exhibited the highest sequence similarity and clustered within the same clade as *B*. *safensis* FO‐36b strain Y175, indicating a close evolutionary relationship (Figure 4). The result was further supported by ANI results, showing 96.09% nucleotide identity with the reference genome of *B. safensis* (Figure 5). The WGS‐based circular phylogram generated using iTOL revealed that isolate S02b is closely related to *B*. *safensis* subsp. *osmophilus* CECT 9344 T and *B*. *safensis* FO‐36b (Figure S3). The genomic map and feature distribution of the bacterial isolate S02b are provided in the Supporting Information (Figure S4 and Table S3).

### 3.4. Analysis of Secondary Metabolite BGCs of Isolate S02b

**Figure 6:**
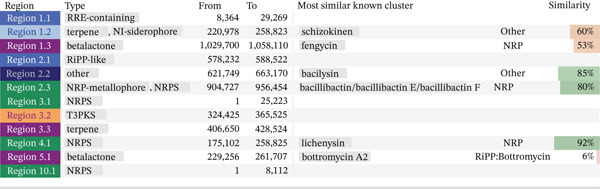
Identified secondary metabolite regions of isolate S02b. Identified BGC clusters showing similarity to schizokinen (60%), fengycin (53%), bacilysin (85%), bacillibactin/bacillibactin E/bacillibactin F (80%), and lichenysin (92%) biosynthetic pathways.

BGC profiling using antiSMASH identified 13 putative BGCs in the genome of isolate S02b. Among these, a terpene and NRPS‐siderophore cluster showed 60% similarity to the schizokinen gene cluster, whereas a *β*‐lactone cluster exhibited 53% similarity to the nonribosomal peptide (NRP) type fengycin cluster. Additionally, an unclassified cluster containing proteins related to secondary metabolites showed 85% similarity to the bacilysin gene cluster. Furthermore, an NRPS and NRP‐metallophore cluster displayed 80% similarity to the bacillibactin/bacillibactin E/bacillibactin F clusters, and another NRPS cluster demonstrated 92% similarity to the lichenysin gene cluster (Figure 6). The predominance of NRPS and posttranslationally modified peptides (RiPP)–like clusters suggests a primary specialization of Isolate S02b in the biosynthesis of peptide‐based secondary metabolites, including antibiotic compounds such as bacilysin and bacillibactin, and antifungal agents like fengycin. Each cluster is color‐coded according to its type, with core biosynthetic, regulatory, and transport genes highlighted in distinct colors (Figure S5).

**Figure 7:**
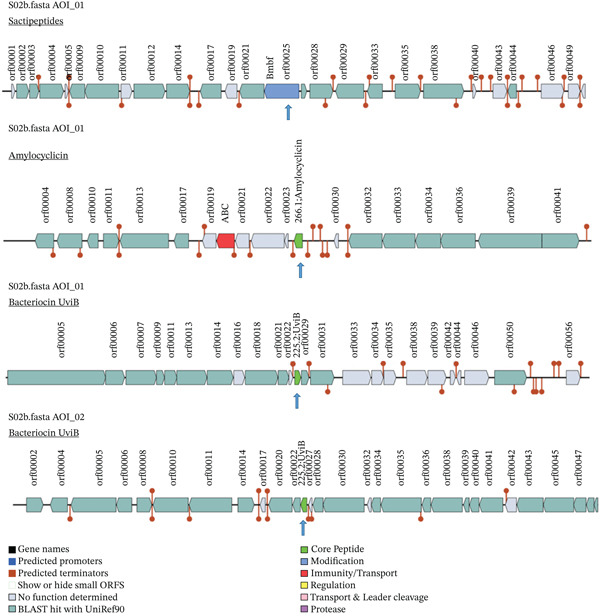
AMPs gene clusters in the AOI, RiPP, and bacteriocin gene clusters of S02b. BAGEL 4.0 analysis of the S02b genome identified one RiPP and three putative bacteriocin gene clusters within the AOIs. The AOI 1 best hit with the sactipeptides synthetase gene cluster (BmbF indicated by blue arrows), circular bacteriocin amylocyclicin (indicated by blue arrows), and bacteriocin UviB protein (UviB as indicated by blue arrows), whereas the AOI 2 best hit to the bacteriocin UviB protein (UviB as indicated by blue arrows).

The BEGEL4 web tool was used to mine the genomic DNA of the bacterial isolate S02b for bacteriocins and RiPPs. The BAGEL 4.0 identified AMPs gene clusters in the area of interest (AOI) and predicted one RiPP and three putative bacteriocin gene clusters of S‐02b. The AOI 1 best hit with the sactipeptide synthetase gene cluster (BmbF), circular bacteriocin amylocyclicin, and bacteriocin UviB protein, whereas the AOI 2 best hit to the bacteriocin UviB protein (Figure 7).

### 3.5. Hemolytic Activity

No hemolytic activity was found on blood agar, indicating the nonhemolytic nature of the extracted antimicrobial compound.

### 3.6. TLC Analysis

**Figure 8:**
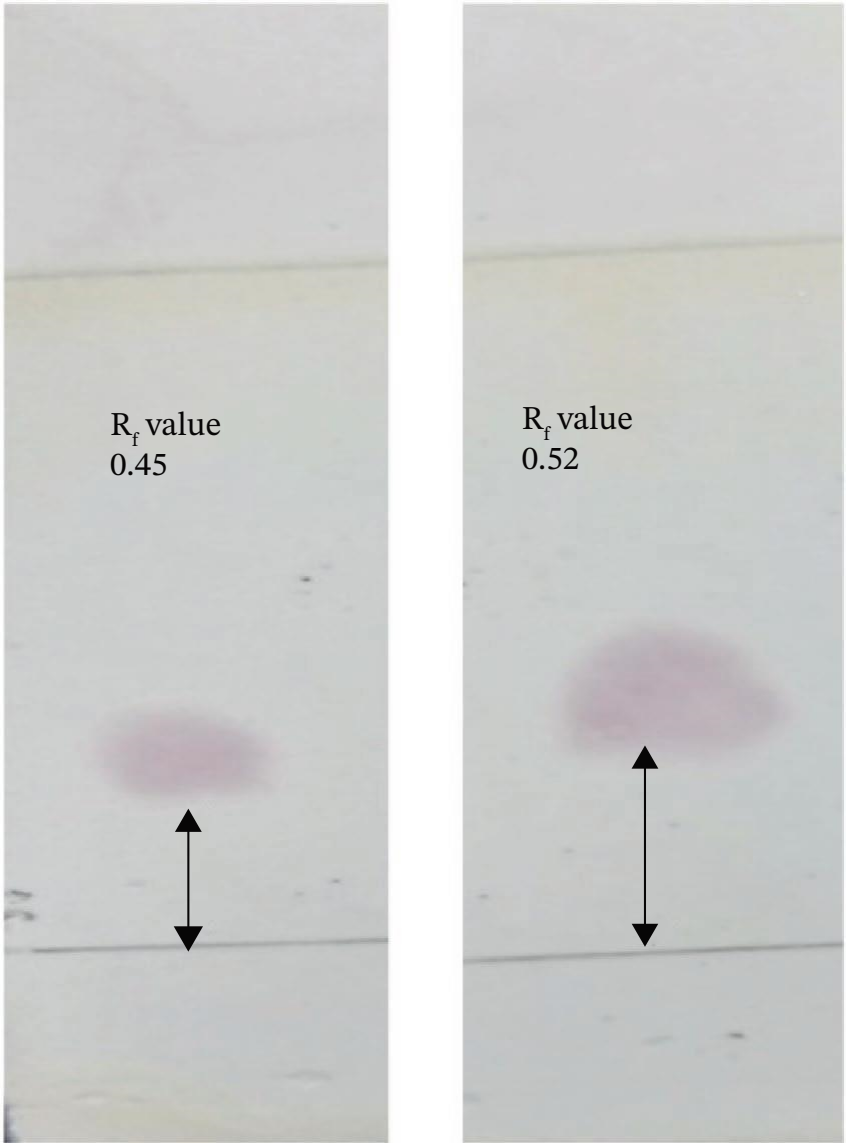
Chromatographic analysis of antimicrobial compounds by thin layer chromatography (TLC) showing *R*_*f*_ value ranging from 0.45 to 0.52.

The extracted antimicrobial compounds were preliminarily identified as single pure compounds by TLC. After measuring the distance traveled by the solvent and the distance traveled by the sample, the *R*_*f*_ value was calculated. The *R*_*f*_ values were noted between 0.45 and 0.52 (Figure 8).

### 3.7. Characterization of the Antimicrobial Compound as APC

After the treatment of the extracted antimicrobial compound with trypsin, reduced ZOI against test host strains was found, indicating the presence of APC in the extracted CFS. The untreated antimicrobial compound was used as the control, and the ZOI was measured (Table 2). Neutral pH (7.4) was observed in the extracted antimicrobial compounds, nullifying the inhibitory effect due to the presence of organic acids.

**Table 2:** Zone of inhibition of trypsin‐treated inhibitory substance against test strains.

Test strain	ZOI of trypsin‐treated APC	ZOI of trypsin‐untreated APC
*B. cereus*	++	+++
*E. coli*	+	+++
*L. monocytogenes*	+	+++

*Note:* (+++): Large ZOI; (++): moderate ZOI; (+): small ZOI.

### 3.8. Spectroscopic Characterization of Antimicrobial Compound

**Figure 9:**
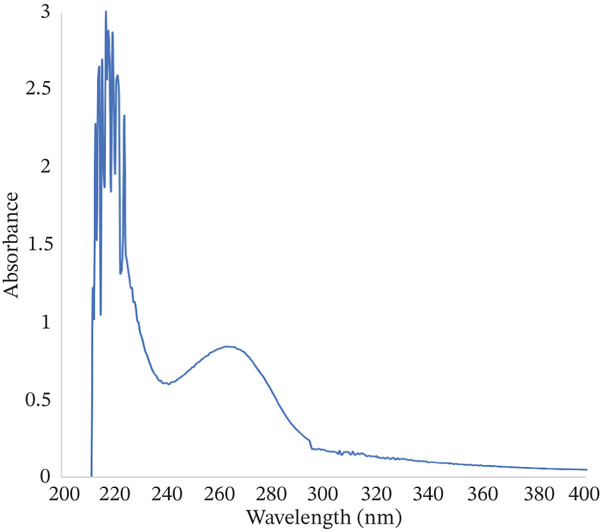
The absorbance band in UV spectra. The strongest absorbance band was at 210–220 nm, which indicates the characteristic absorption of peptides and the presence of protein.

There is no significant absorption peak above 300 nm in UV spectra (Figure 9). The intense peak at 210–220 nm indicates the characteristic absorption of peptides and the presence of protein in the extracted CFS. The absorption peak was found near 270 nm, which indicated the probable presence of aromatic amino acids (tryptophan, tyrosine, or phenylalanine) in the protein.

### 3.9. Stability of the Extracted APC

The stability of extracted APC from isolate S02b was evaluated against different temperatures, pH, surfactants, organic solvents, and food‐grade metal salts. The ZOI of APC at 30°C after 72 h was used as a control. The extracted APC was heat‐stable up to 60°C, and then its activity gradually decreased with increasing temperature. It showed a 50% loss of activity at 100°C. APC activity was stable over pH 6.5–10; however, a slight loss of activity was observed below pH 5. APC was found to be stable in the presence of surfactants, including Triton X‐100 (95%), Tween‐20 (81%), and SDS (80%); however, activity was fully diminished in the presence of Tween‐80. APC was active with all food‐grade metals used in this study; however, in the presence of zinc sulfate, it retained about 96% of its activity. The antimicrobial activity of extracted APC was observed in the presence of all organic solvents and compounds used in this study (100%), except n‐hexane and dimethyl sulfonate. APC was active in all organic acids (100%) used except citric acid (Table 3).

**Table 3:** Stability of the antimicrobial activity of APC at different parameters.

Treatment	% Activity
**Temperature (exposed for 15 min)**	
50°C	100
60°C	85
70°C	85
80°C	82
90°C	71
100°C	50
**pH**	
3	93
5	93
6.5	100
8	100
10	100
**Surfactants (1.0% v/v)**	
SDS	80
Tween 20	81
Tween 80	0
Triton X‐100	95
**Food-grade metal salts (1.0 mg/mL)**	
CuSO_4_	100
MgSO_4_	100
MnCl_2_	100
AgNO_3_	100
FeSO_4_	100
CaCl_2_	100
ZnSO_4_	96
**Organic solvents (50% v/v)**	
Ethyl alcohol	100
Methanol	100
Chloroform	100
Isopropanol	100
Phenol	100
DMSO	100
EDTA (2.5 and 50 mg/mL)	100
n‐hexane	0
Dimethyl sulfonate	0
**Organic acids (10.0 mg/mL)**	
Tartaric acid	100
Lactic acid	100
Citric acid	0

### 3.10. SEM Analysis

**Figure 10:**
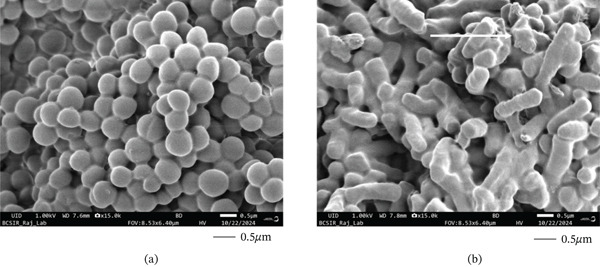
Scanning electron microscopy images. (a) Control. (b) Treated with CFS containing APC. The rupture in cell morphology of the test pathogen, *S. aureus,* treated with extracted CFS containing APC was observed.

The outcome of the cell morphology of the test pathogen (*S. aureus*) treated with extracted CFS containing APC was examined by SEM, and changes or any rupture in the cell morphology were observed and shown in Figure 10.

## 4. Discussion

Many gram‐positive bacteria, including *Bacillus,* in complex environments, produce antimicrobial compounds [45]. Habitats like the soil‐rhizosphere provide a competitive environment, producing antimicrobial compounds by multiple microbes that exhibit a selective spectrum of inhibition [46]. Screening and characterization of novel APCs, such as AMPs, have attracted the attention of many researchers due to their potential applications in the therapeutic and food industry [47]. Studies found that strains of *Bacillus* produce a diverse array of antimicrobial compounds which are active against food‐spoilage and pathogenic bacteria [48, 49].

The antimicrobial activity of inhibitory compounds can be evaluated by following the agar well diffusion assay, spot‐on‐lawn assay, turbidimetric assay, ELISA, and radiometry [50]. APCs like AMPs can play a vital role in the food industry, where they are incorporated into complex food environments during food processing [51].

Our study demonstrated that *Bacillus* spp. isolated from rhizosphere soil samples exhibited potential antimicrobial activity. In a previous study, *Bacillus* spp. exhibited antimicrobial activity predominantly against gram‐positive bacteria rather than gram‐negative organisms, likely due to differences in cell wall composition [52] [53]. Gram‐negative bacteria possess lipopolysaccharides (LPS) in the outer membrane, which act as a surface barrier capable of hindering the penetration of certain lipopeptides, thereby reducing their interaction with the cytoplasmic membrane [54]. Additionally, antimicrobials derived from *Bacillus*, such as bacitracin and mersacidin, inhibit the biosynthesis of peptidoglycan, an essential component of the bacterial cell wall [55]. As gram‐positive bacteria have a thick, exposed peptidoglycan layer, they are more susceptible to these agents than gram‐negative bacteria, as they possess a thinner peptidoglycan layer that is further protected by an outer membrane, resulting in reduced susceptibility [56]. However, in the present study, *Bacillus* spp. were found to produce APCs with antimicrobial activity against both gram‐negative and gram‐positive bacteria, suggesting the possibility that these compounds may possess dual mechanisms of action, involving the disruption of both the bacterial cell wall and the cell membrane. In our study, three strains of *Bacillus*, namely *B*. *safensis* (*n* = 2) and *B*. *pseudomycoides* (*n* = 1), produced antimicrobial compounds that exhibited antimicrobial activity against gram‐negative test strains *E. coli, P. aeruginosa*, and *S. dysenteriae*. Interestingly, the study strain *B. safensis* exhibited antimicrobial activity against a multidrug‐resistant gram‐negative clinical strain of *E. coli*. In the preliminary screening of 27 *Bacillus* isolates, 5 (5/27; 18%) produced inhibitory compounds with antibacterial activity. After optimization of media, temperature, and pH, three study isolates (*n* = 3/5) showed enhanced antimicrobial compounds production at 30°C, pH 7. Antimicrobial compound production was also observed at 30°C, pH 5.5, and the compound was effective against *L. monocytogenes* and *B. cereus*. The production was effective against *S. aureus* and *B. cereus* at 37°C and pH 7 after 120 h of incubation; however, production was not detected thereafter. It was observed that antimicrobial compound production gradually increased with prolonged incubation time, then reached a peak and subsequently decreased. The decline in antimicrobial compound production was accompanied by reduced antimicrobial activity, as evidenced by a decrease in the measured ZOI around the test strains. The production of APCs starts after 3 h and reaches a maximum during the early stationary phase [57]. This study observed that the highest level of APCs production was achieved after a 72‐h incubation period, when the culture of the study strain was considered to be in the stationary phase. However, the relationship between a longer incubation period and better APC production was not statistically significant. On the other hand, APC production was higher at neutral pH (pH 7) than at acidic pH conditions, and this difference was statistically significant. However, the higher APC production at pH 7 compared with alkaline pH was not statistically significant. If the number of samples and study isolates could be increased in this study, the actual association among all the parameters used for better APCs production could have been revealed. In biotechnology research, *Bacillus* spp. are significant due to their ability to produce structurally diverse arrays of secondary metabolites, including antimicrobial compounds [12, 14, 58]. WGS revealed isolate S02b harbors BGCs showing similarity to schizokinen (60%), fengycin (53%), bacilysin (85%), bacillibactin variants (80%), and lichenysin (92%) pathways, indicating its specialization in the biosynthesis of peptide‐based secondary metabolites, including antibiotics (bacilysin and bacillibactin) and antifungal (fengycin) compounds. Furthermore, WGS revealed AMP gene clusters in the AOIs, including one RiPP and three putative bacteriocin clusters in isolate S‐02b. AOI 1 showed homology to sactipeptide synthetase (BmbF), circular bacteriocin amylocyclicin, and bacteriocin UviB protein clusters, whereas AOI 2 corresponded to a bacteriocin UviB protein cluster. For evaluation of the purity of APC, TLC revealed that one type of protein was present in the samples, exhibiting single spot on the TLC plate. Extracted APCs from *B*. *safensis* (S02b and S03b) and *B*. *pseudomycoides* (S04b) showed significant ZOI against gram‐positive (*S. aureus* and *L. monocytogenes*) and gram‐negative (*E. coli, P. aeruginosa,* and *S. dysenteriae)* test host strains; moreover, they had antibacterial activity against multidrug‐resistant clinical isolate *E. coli,* which was resistant to 13 different antibiotics, including fourth‐generation cephalosporin, cefepime. The nature of the extracted antimicrobial compound was found to be proteinaceous, as it was treated with the proteolytic enzyme trypsin, and the antimicrobial activity of the compound was reduced. Moreover, the neutral pH of CFS indicated no acid production. The increased ZOI produced by the antimicrobial compounds extracted from *B*. *safensis* and *B*. *pseudomycoides* under optimized conditions (30°C, pH 7, and 72 h), together with the reduced activity after enzymatic treatment and the absence of organic acid production, indicates that the antimicrobial effect is due to proteinaceous antimicrobial compounds. A study revealed that peptide bond absorption occurs in the UV range of 180–220 nm. If aromatic side chains (tryptophan, tyrosine, and phenylalanine) were present, the absorption bands shifted to the near‐UV region of 250–300 nm [41]. The intense absorption peak near 280 nm indicated the presence of tryptophan, whereas a comparatively weaker absorbance at 276 nm indicated the presence of the phenol side chain of tyrosine, and the weakest absorbance, resulting from the benzene side chain of phenylalanine, was in the 250–270 nm [41]. In our study, the strongest peak was observed at 210–220 nm, indicating characteristic peptide absorption and the presence of protein in the extracted CFS. The absorption peak was located near 270 nm, indicating the probable presence of aromatic amino acids (tryptophan, tyrosine, or phenylalanine) in the extracted APCs. *B. safensis* contains genes encoding several putative AMPs. The antimicrobial compounds produced by *B. safensis* could play a significant role in preventing bacterial colonization by noncommensal microorganisms [59]. In our study, APC extracted from *B. safensis* was heat‐stable up to 60°C. As no hemolysis was found on blood agar, the extracted APC is considered nonhemolytic. Moreover, its stability under varying pH, surfactants, organic solvents, organic acids, and food‐grade metal salts suggests that this APC could be a promising candidate for use as a food additive. Therefore, our study strain, *B. safensis*, produces APCs that could potentially be applied in the clinical and food industries. *B. safensis* has been reported as an essential bacterial strain for industrial and scientific studies as it has antifungal activity [60], degrading ability of petroleum pollutants [61], and also helps in the promotion of plant growth [62]. In our study, another strain, *B*. *pseudomycoides,* showed antimicrobial activity against gram‐negative bacteria. A report stated novel lantibiotic pseudomycoicidin was produced by *B*. *pseudomycoides,* having antimicrobial activity against gram‐positive bacteria [63]. Further research will be conducted to identify the APC, determine its minimum inhibitory concentration (MIC), and assess its tolerance to bile salts and gastric acidic conditions in the future.

## 5. Conclusions

In this study, we isolated APC‐producing *B*. *safensis* and *B*. *pseudomycoides* from rhizosphere soil. The APCs exhibited strong antimicrobial activity against both gram‐positive and gram‐negative bacteria, including an MDR clinical isolate of *E*. *coli*. These findings suggest that APCs derived from *B. safensis* and *B. pseudomycoides* could serve as promising alternatives to conventional antibiotics for combating MDR pathogens. Moreover, the results highlight the potential of these APCs as novel bioactive scaffolds for future therapeutic and food preservation applications.

## Funding

No funding was received for this manuscript.

## Conflicts of Interest

The authors declare no conflicts of interest.

## Supporting information


**Supporting Information** Additional supporting information can be found online in the Supporting Information section. Table S1: The geographic locations of the soil sampling sites and their corresponding soil textural classifications. Representative images of the morphological and biochemical characterization of the study isolates are presented in Figures S1 and S2. The biochemical characteristics of the bacterial isolates are summarized in Table S2. Whole‐genome sequence (WGS)–based phylogenomic analysis of isolate S02b is presented in Figure S3, whereas its genomic features are summarized in Table S3. A circular genome map of isolate S02b, illustrating coding sequences (CDSs), rRNA, tRNA, ncRNA, GC content, GC skew, and CRISPR elements, is shown in Figure S4. The biosynthetic gene clusters (BGCs) identified in the genome of isolate S02b, including RiPP recognition element (RRE), terpene, NRPS‐siderophore, *β*‐lactone, RiPP‐like, bacilysin, NRP‐metallophore, NRPS, terpene‐precursor, and T3PKS clusters, are presented in Figure S5.

## Data Availability

The data that support the findings of this study are available in the Supporting Information of this article.
