# Supplementary material for: Isolation, Characterization, and Optimization of Antimicrobial Proteinaceous Compounds Produced by Bacillus safensis Isolated From Rhizosphere Soil Samples in Bangladesh
Source: Int J Microbiol. 2026 Jul 1;2026:3189187. doi: 10.1155/ijm/3189187 (PMC13323842; doi:10.1155/ijm/3189187)
Supplement: Supplementary file 1 — Supporting Information Additional supporting information can be found online in the Supporting Information section. Table S1: The geographic locations of the soil sampling sites and their corresponding soil textural classifications. Representative images of the morphological and biochemical characterization of the study isolates are presented in Figures S1 and S2. The biochemical characteristics of the bacterial isolates are summarized in Table S2. Whole‐genome sequence (WGS)–based phylogenomic analysis of isolate S02b is presented in Figure S3, whereas its genomic features are summarized in Table S3. A circular genome map of isolate S02b, illustrating coding sequences (CDSs), rRNA, tRNA, ncRNA, GC content, GC skew, and CRISPR elements, is shown in Figure S4. The biosynthetic gene clusters (BGCs) identified in the genome of isolate S02b, including RiPP recognition element (RRE), terpene, NRPS‐siderophore, β‐lactone, RiPP‐like, bacilysin, NRP‐metallophore, NRPS, terpene‐precursor, and T3PKS clusters, are presented in Figure S5. [file IJM-2026-3189187-s001.docx]

**Supplementary Info**

**Supplementary Information Table 1: Geographic locations of soil sampling sites and associated soil textural classifications.**

| Soil samples from  different sites | Textural classification of soil | Location Coordinates (Latitude, Longitude) |
| --- | --- | --- |
| Site 1 | Silty clay | 23.876583° N, 90.267389° E |
| Site 2 | Silty clay | 23.876194° N, 90.268833° E |
| Site 3 | Silty clay | 23.877917° N, 90.262056° E |
| Site 4 | Silty clay | 23.878722° N, 90.262028° E |
| Site 5 | Silty Loam | 23.876556° N, 90.246944° E |
| Site 6 | Silty Loam | 23.876500° N, 90.246500° E |
| Site 7 | Silty Loam | 23.876917° N, 90.247222° E |
| Site 8 | Silty Loam | 23.877167° N, 90.245472° E |


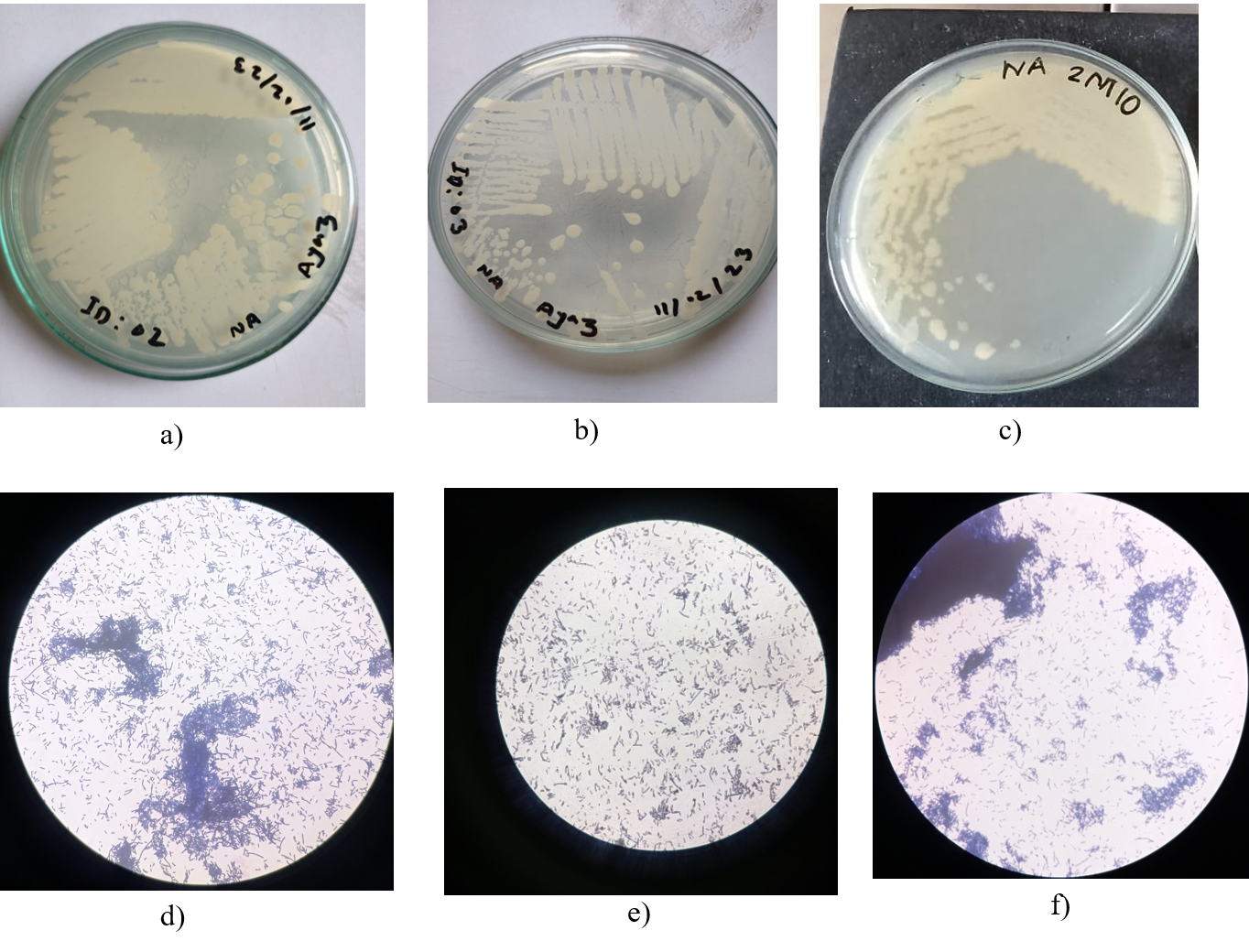


**Supplementary Information Fig. 1:** Representative image of morphological and Gram-staining of study isolates. a) Isolate S02b b) Isolate S03b c) Isolate S04b on nutrient agar (NA); d) Isolate S02b e) Isolate S03b f) Isolate S04b were Gram-positive, rod under microscopic observation.


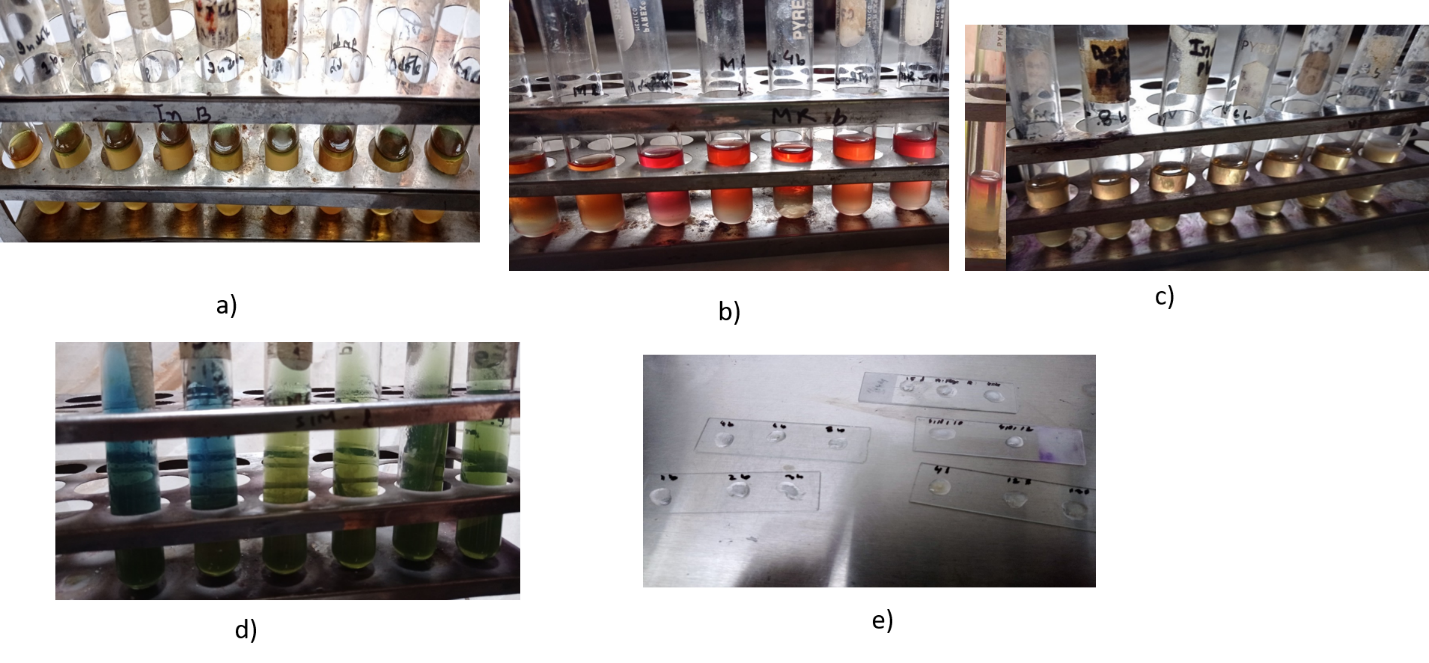


**Supplementary Information Fig. 2:** Representative image of biochemical test of study isolates. a) Indole test b) MR test c) VP test, d) Citrate utilization test e) catalase test. All presumptive isolates were indole-negative, catalase-positive, and capable of starch hydrolysis. Eighty-eight percent of isolates were MR-positive, while all were VP-negative. Fifteen percent isolates were capable of citrate utilization.

**Supplementary Information Table 2: Biochemical profiles of the bacterial isolates included in this study.**

| Isolate | Indole | MR | VP | Citrate utilization | Catalase | Starch Hydrolysis |
| --- | --- | --- | --- | --- | --- | --- |
| 1 b | - | + | - | - | + | + |
| 2 b | - | - | - | - | + | - |
| 3 b | - | - | - | - | + | - |
| 4 b | - | + | - | - | + | - |
| 5 b | - | + | - | - | + | - |
| 6 b | - | + | - | - | + | + |
| 7 b | - | + | - | - | + | - |
| 8 b | - | - | - | - | + | + |
| 9 b | - | + | - | - | + | - |
| 10 b | - | + | - | + | + | + |
| 11 b | - | + | - | + | + | - |
| 12 b | - | + | - | + | + | - |
| 13 b | - | + | - | + | + | - |
| 14 b | - | + | - | - | + | - |
| 15 b | - | + | - | - | + | - |
| 16 b | - | + | - | - | + | - |
| 17 b | - | + | - | - | + | - |
| 18 b | - | + | - | - | + | - |
| 19 b | - | + | - | - | + | - |
| 20 b | - | + | - | - | + | + |
| 21 b | - | + | - | - | + | - |
| 22 b | - | + | - | - | + | - |
| 23 b | - | + | - | - | + | - |
| 24 b | - | + | - | - | + | - |
| 25 b | - | + | - | - | + | - |
| 26 b | - | + | - | - | + | - |
| 27 b | - | + | - | - | + | - |


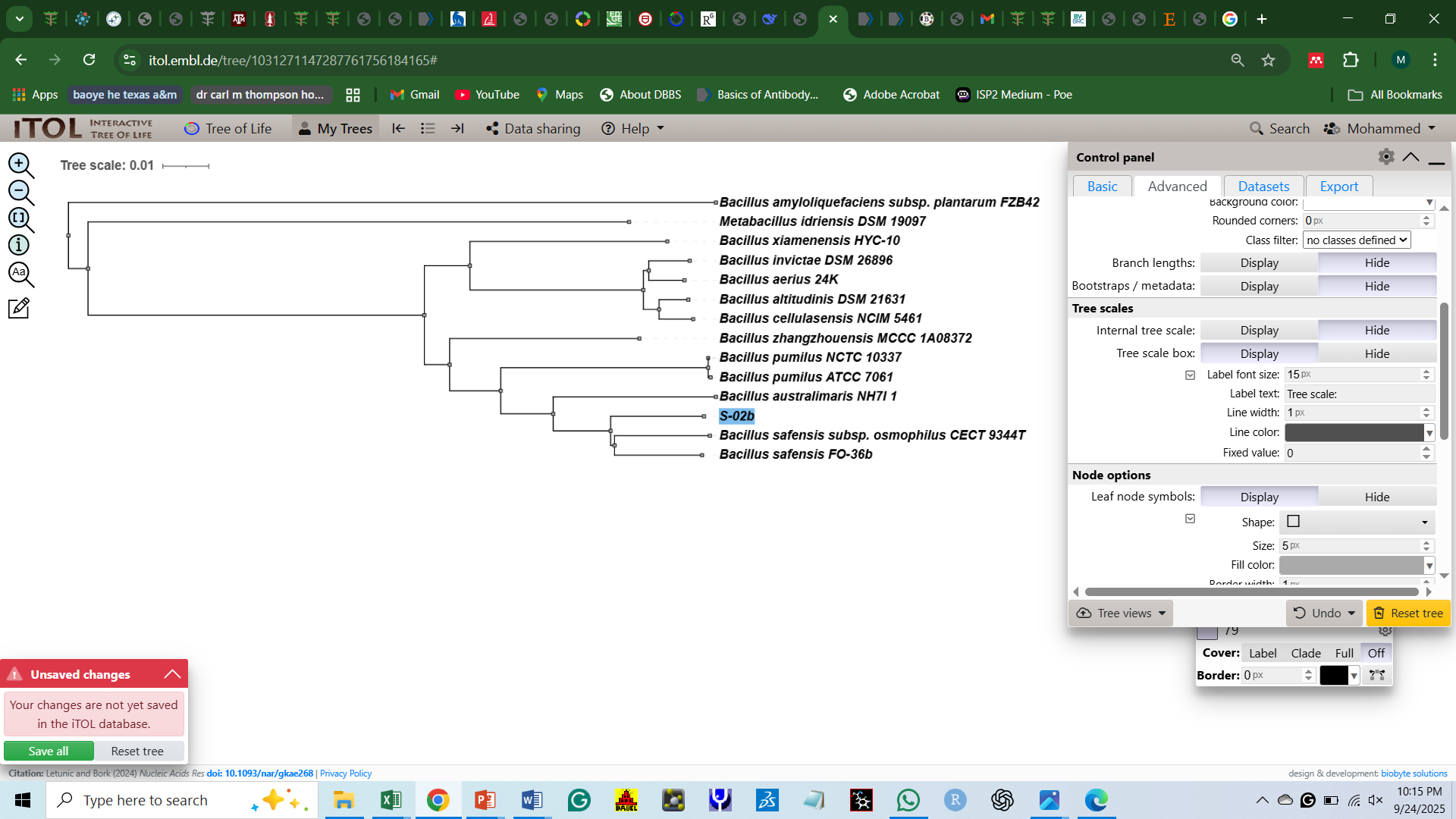


**Supplementary Information Fig. 3:** **WGS-based Circular Phylogram by iTOL.** Sequence alignment and construction of the phylogenetic tree revealed that the isolate is very closely related to *Bacillus safensis* strain subsp., *osmophilus* CECT 9344T, and *Bacillus safensis* FO-36b.

**Supplementary Information Table 3;** **Genomic feature distribution of the bacterial isolate S02b**

| Contigs | 23 |
| --- | --- |
| GC Content | 41.4 |
| Contig L50 | 2 |
| Genome Length | 3,760,616 bp |
| Contig N50 | 982,520 |
| CDS | 3,939 |
| tRNA | 58 |
| rRNA | 3 |
| Hypothetical proteins | 828 |
| Proteins with functional assignments | 3,111 |
| Proteins with EC number assignments | 940 |
| Proteins with GO assignments | 784 |
| Proteins with Pathway assignments | 698 |
| Proteins with PATRIC genus-specific family (PLfam) assignments | 3,701 |
| Proteins with PATRIC cross-genus family (PGfam) assignments | 3,802 |


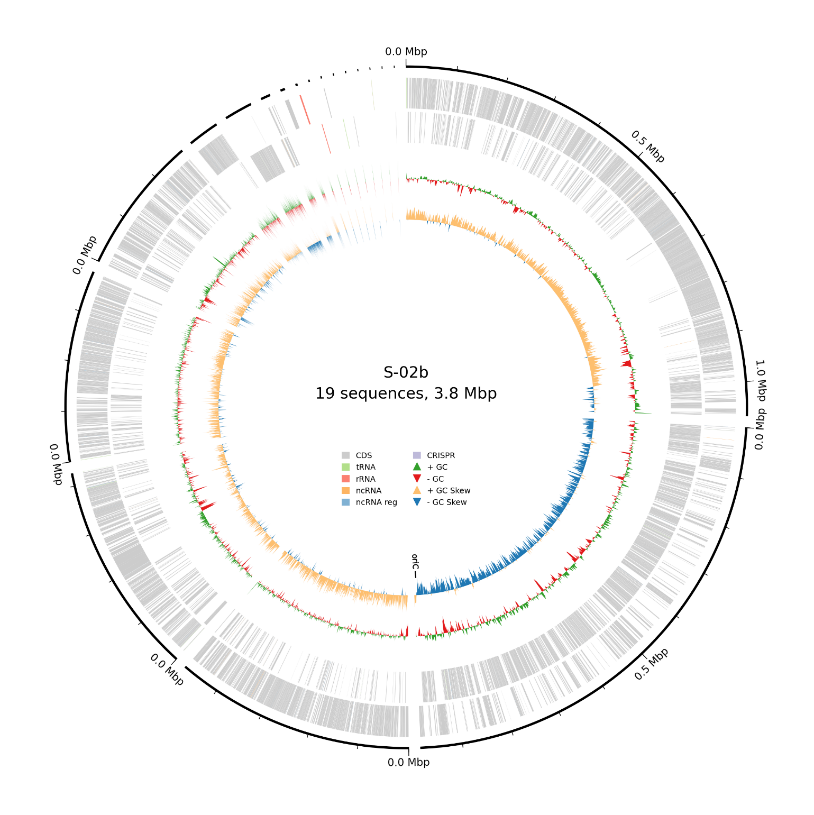


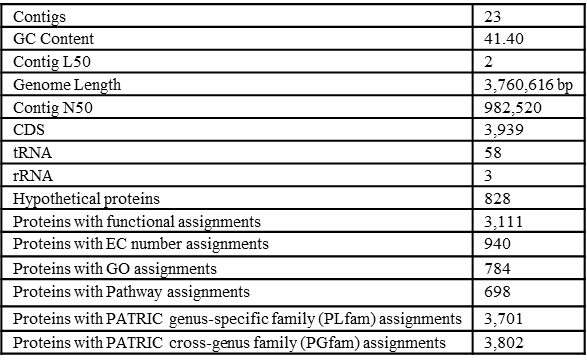


**Supplementary Information Fig. 4**: Genomic map of S02b. From the outermost to the innermost circle, the map displays coding sequences (CDSs), rRNA, tRNA and ncRNA, GC content, GC skew, and CRISPR. The sixth and seventh circles illustrate the mobile genetic elements distribution on the positive and negative strands.


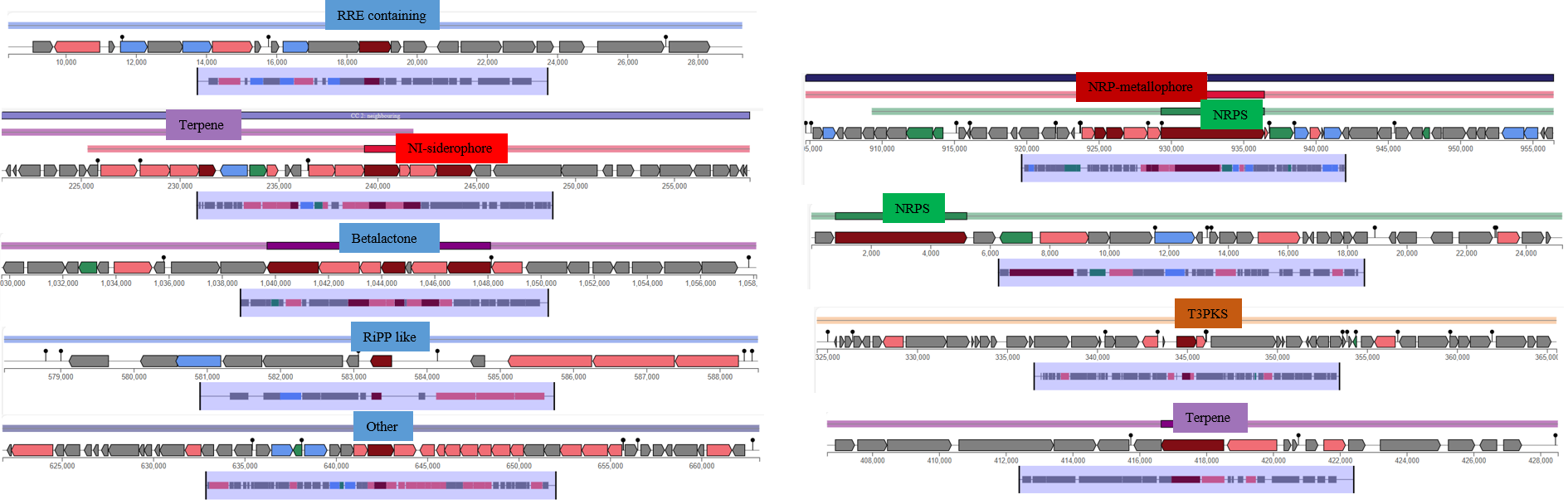


**Supplementary Information Fig. 5: Biosynthetic gene clusters (BGCs) identified in the genome of isolate S02b using antiSMASH.** The identified clusters include RiPP recognition element (RRE), terpene, NRPS-siderophore, β-lactone, RiPP-like, other (bacilysin), NRP-metallophore, NRPS, terpene-precursor, and T3PKS. Each cluster is color-coded according to its type, with core biosynthetic, regulatory, and transport genes highlighted in different colors.
